# Supplementary figures and images for: Decrease of Nibrin expression in chronic hypoxia is associated with hypoxia-induced chemoresistance in some brain tumour cells
Source: BMC Cancer. 2019 Apr 3;19:300. doi: 10.1186/s12885-019-5476-9 (PMC6446413; doi:10.1186/s12885-019-5476-9)

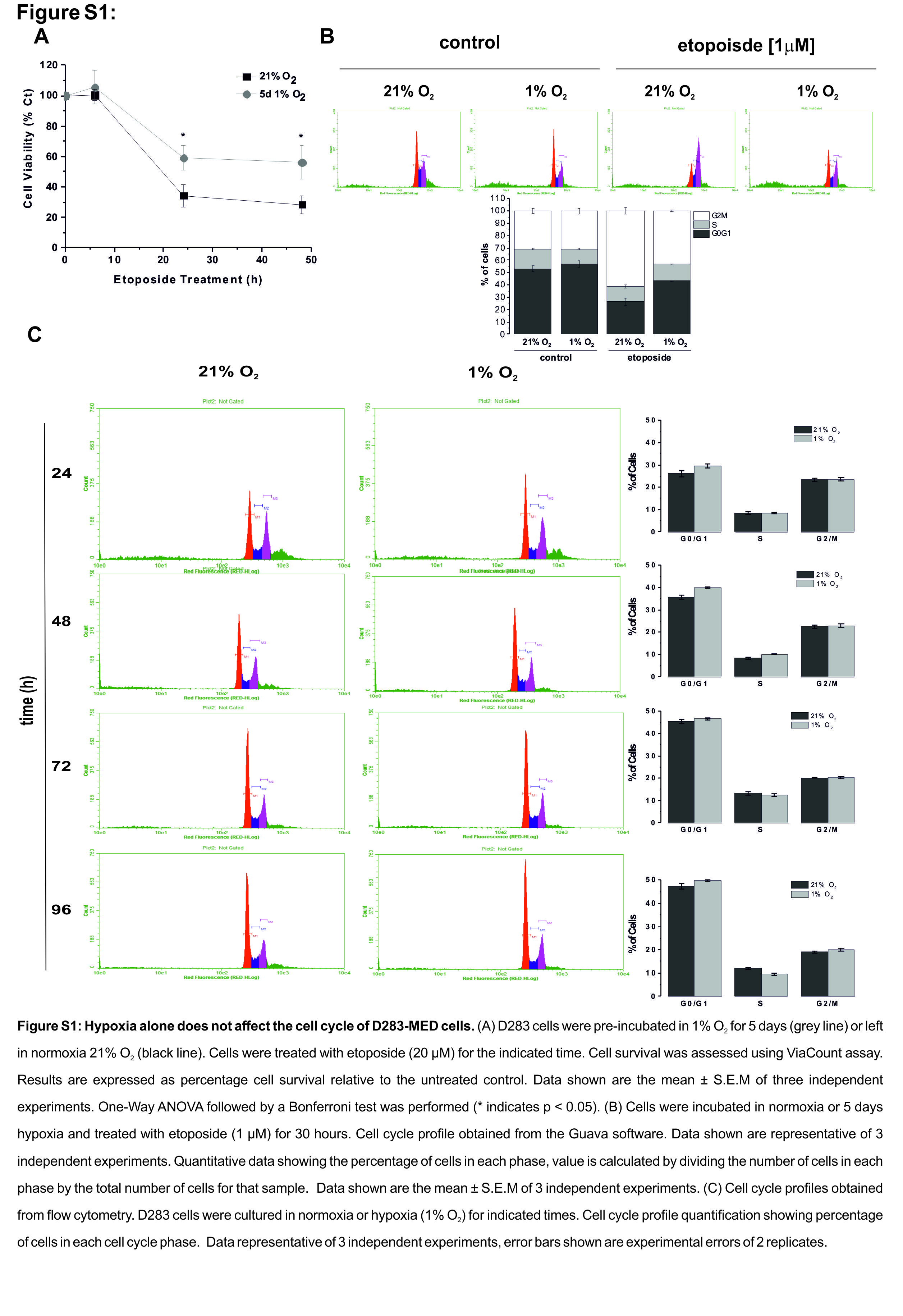

Supplement: Supplementary file 1 — Figure S1. Hypoxia alone does not affect the cell cycle of D283-MED cells. D283-MED cells were pre-incubated in 1% O2 for 5 days or left in normoxia 21% O2. Cells were treated with etoposide (20 μM) for the indicated time. Cell survival was assessed using ViaCount assay. (B) Cells were incubated in 21% O2 or 5 days in 1% O2 and treated with etoposide (1 μM) for 30 h. Cell cycle profile obtained from the Guava software. Quantitative data showing the percentage of cells in each phase, value is calculated by dividing the number of cells in each phase by the total number of cells for that sample. (C) Cell cycle profiles obtained from flow cytometry. D283-MED cells were cultured in normoxia or hypoxia (1% O2) for indicated times. Cell cycle profile quantification showing percentage of cells in each cell cycle phase. (JPG 3287 kb) [file 12885_2019_5476_MOESM1_ESM.jpg]

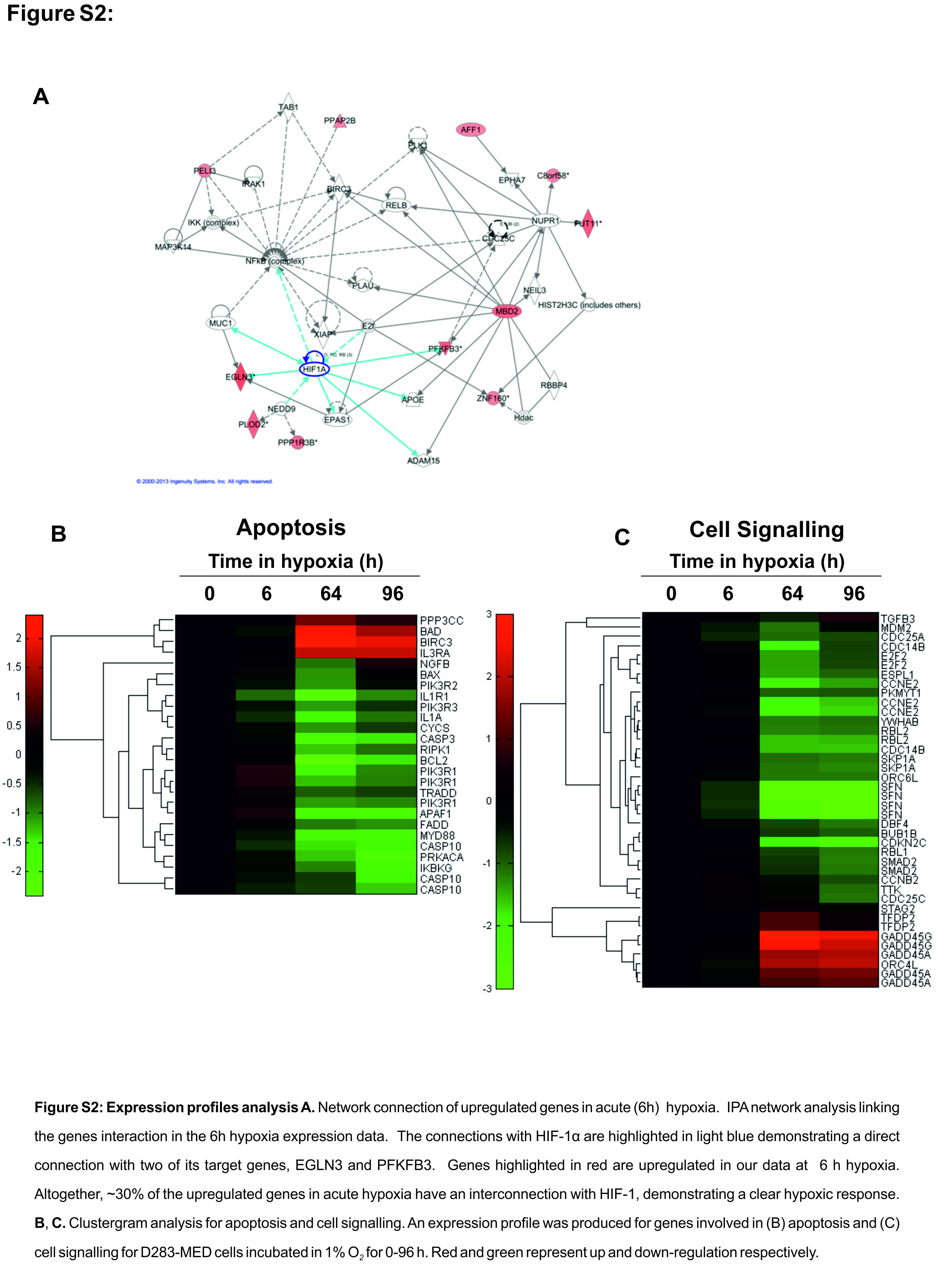

Supplement: Supplementary file 2 — Figure S2. Expression profile analysis (A) Network connection of upregulated genes in acute (6 h) hypoxia. IPA network analysis linking the genes interaction in the 6 h hypoxia expression data. The connections with HIF-1α are highlighted in light blue demonstrating a direct connection with two of its target genes, EGLN3 and PFKFB3. Genes highlighted in red are upregulated in our data at 6 h hypoxia. Altogether, ~ 30% of the upregulated genes in acute hypoxia have an interconnection with HIF-1, demonstrating a clear hypoxic response. (B, C) Clustergram analysis for apoptosis and cell signalling. An expression profile was produced for genes involved in (B) apoptosis and (C) cell signalling for D283-MED cells incubated in 1% O2 for 0–96 h. (JPG 3108 kb) [file 12885_2019_5476_MOESM2_ESM.jpg]

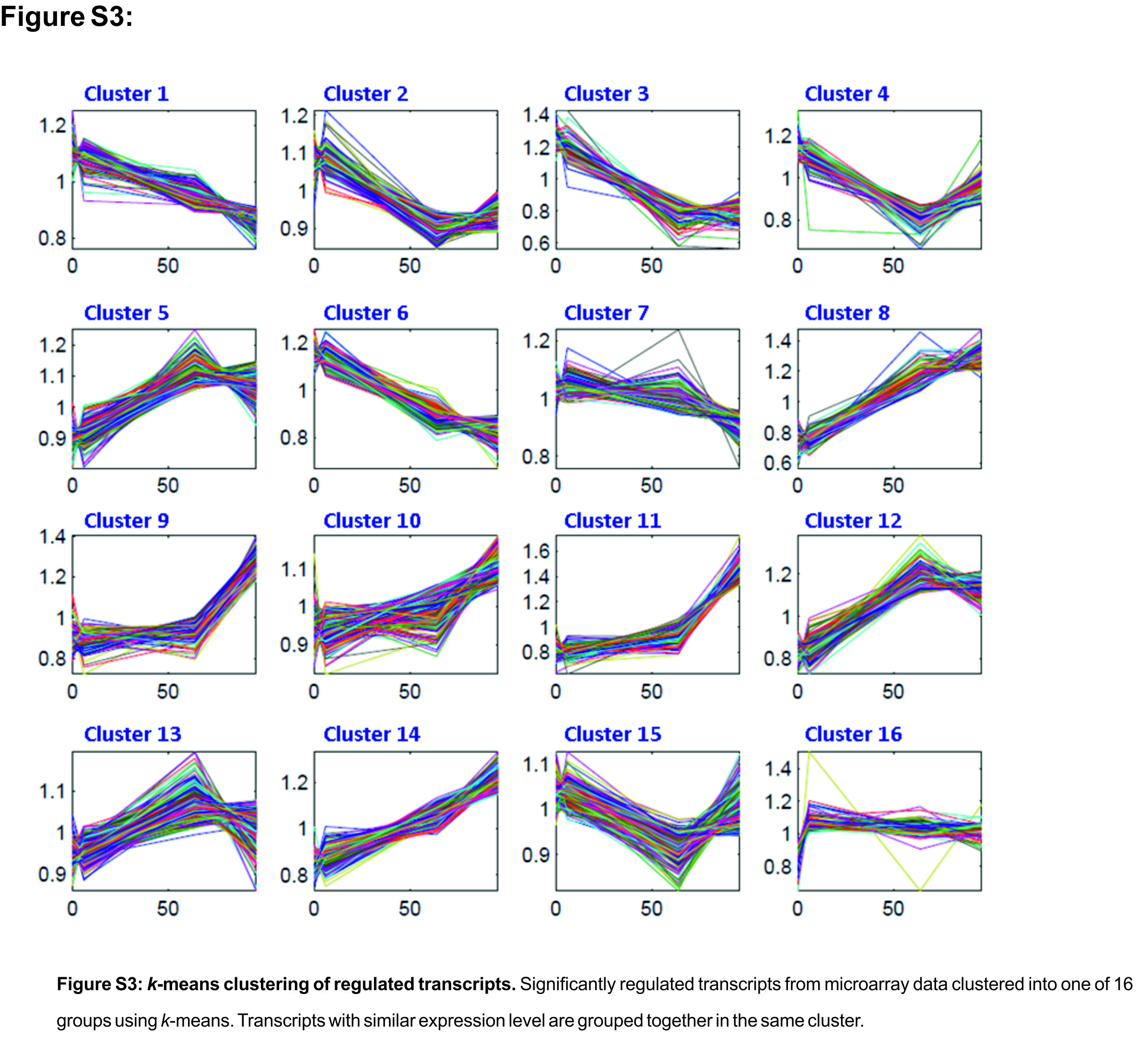

Supplement: Supplementary file 3 — Figure S3. k-means clustering of regulated transcripts. Significantly regulated transcripts from microarray data clustered into one of 16 groups using k-means. Transcripts with similar expression level are grouped together in the same cluster. (JPG 3008 kb) [file 12885_2019_5476_MOESM3_ESM.jpg]

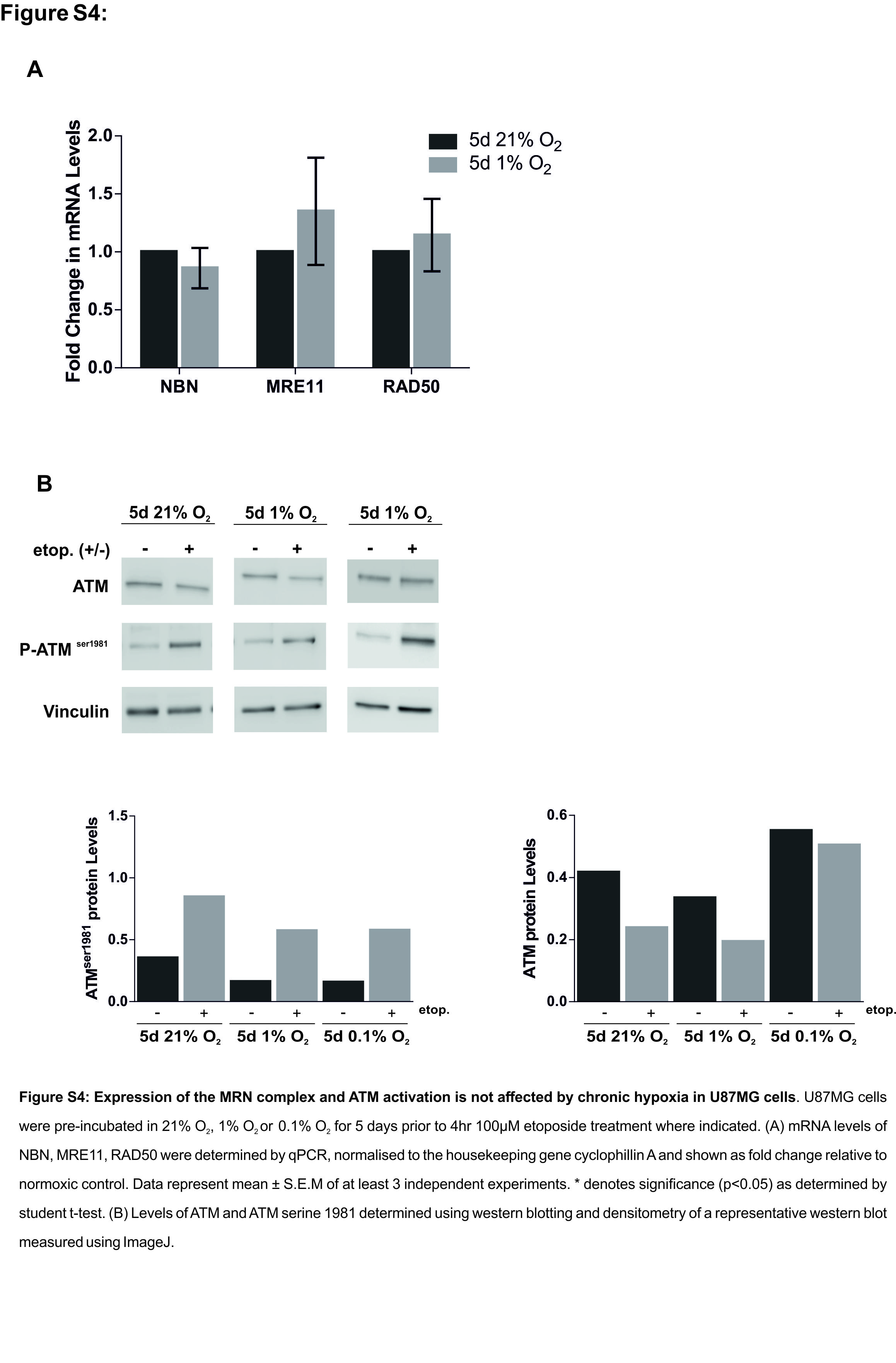

Supplement: Supplementary file 4 — Figure S4. Expression of the MRN complex and ATM activation are not affected by chronic hypoxia in U87MG cells. U87MG cells were pre-incubated in 21% O2, 1% O2 or 0.1% O2 for 5 days prior to 4 h 100 μM etoposide treatment where indicated. (A) mRNA levels of NBN, MRE11, RAD50 were determined by qPCR, normalised to the housekeeping gene cyclophillin A and shown as fold change relative to normoxic control. (B) Levels of ATM and ATM serine 1981 determined using western blotting and densitometry of a representative western blot measured using ImageJ. (JPG 2679 kb) [file 12885_2019_5476_MOESM4_ESM.jpg]

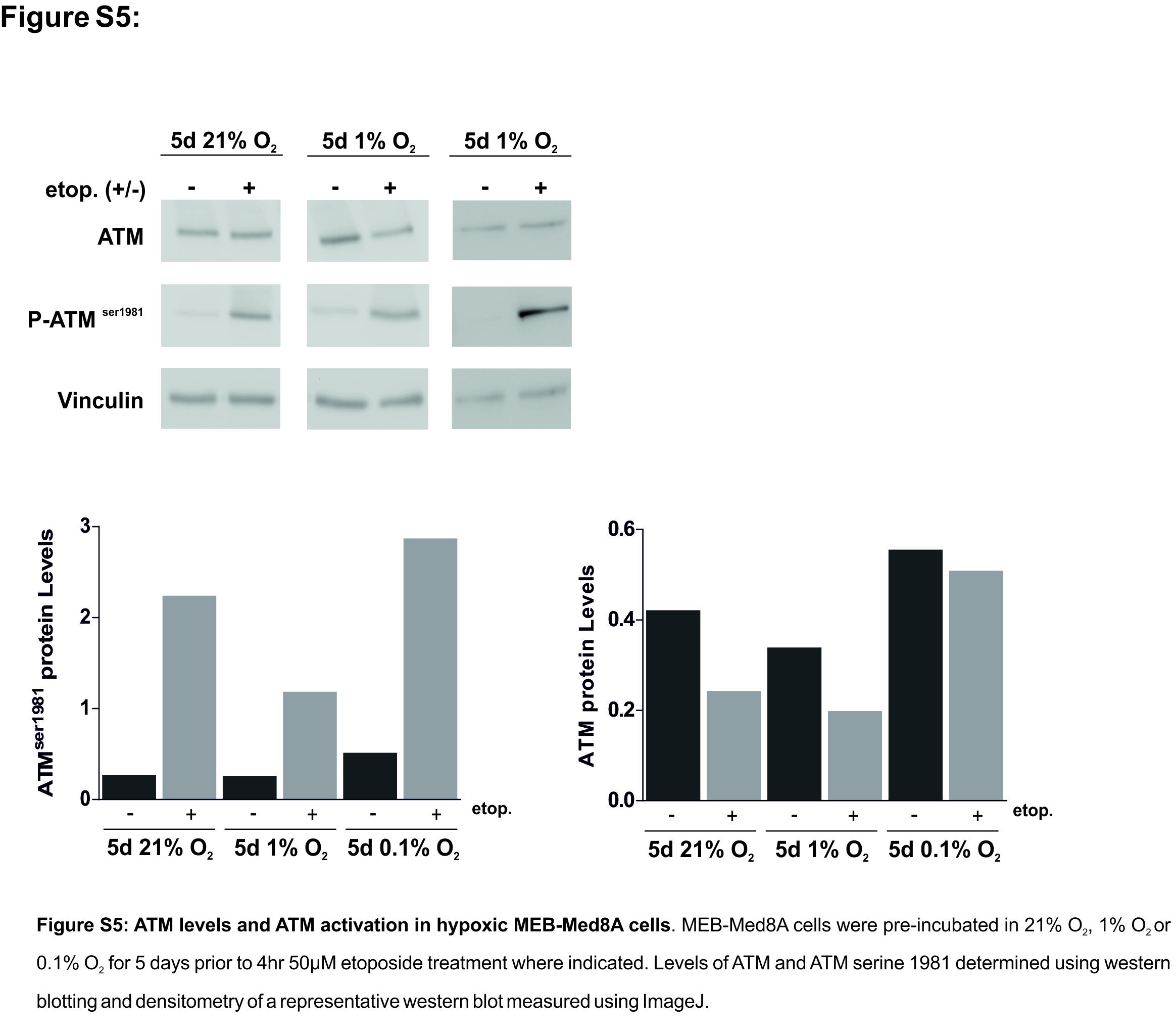

Supplement: Supplementary file 5 — Figure S5. ATM levels and ATM activation in hypoxic MEB-Med8A cells. MEB-Med8A cells were pre-incubated in 21% O2, 1% O2 or 0.1% O2 for 5 days prior to 4 h 50 μM etoposide treatment where indicated. Levels of ATM and ATM serine 1981 determined using western blotting and densitometry of a representative western blot measured using ImageJ. (JPG 2299 kb) [file 12885_2019_5476_MOESM5_ESM.jpg]

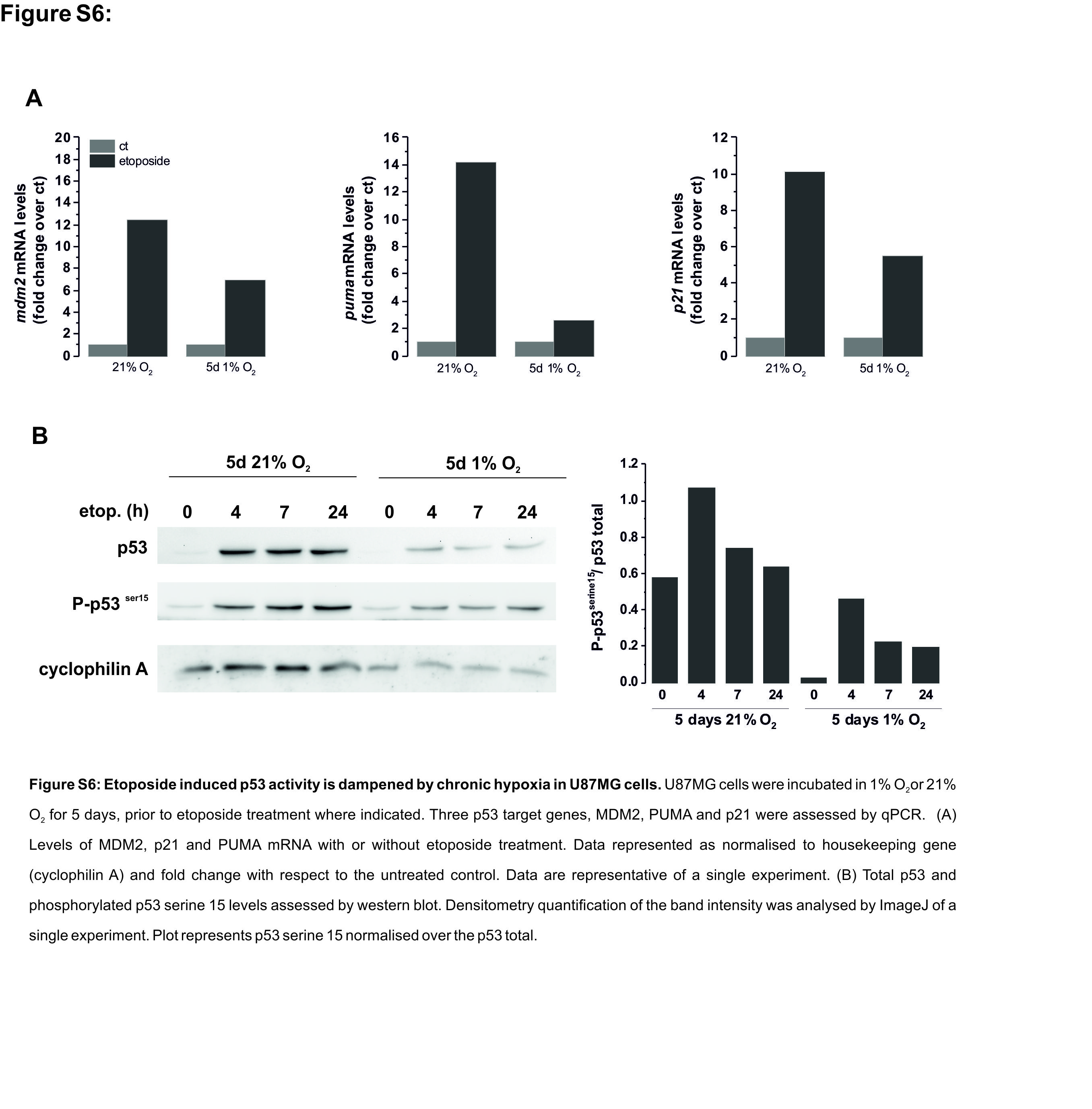

Supplement: Supplementary file 6 — Figure S6. Etoposide induced p53 activity is dampened by chronic hypoxia in U87MG cells. U87MG cells were incubated in 1% O2 or 21% O2 for 5 days, prior to etoposide treatment where indicated. Three p53 target genes, MDM2, PUMA and p21 were assessed by qPCR. (A) Levels of MDM2, p21 and PUMA mRNA with or without etoposide treatment. Data represented as normalised to housekeeping gene (cyclophilin A) and fold change with respect to the untreated control. Data are representative of a single experiment. (B) Total p53 and phosphorylated p53 serine 15 levels assessed by western blot. Densitometry quantification of the band intensity was analysed by ImageJ of a single experiment. Plot represents p53 serine 15 normalised over the p53 total. (JPG 2722 kb) [file 12885_2019_5476_MOESM6_ESM.jpg]
